# Lipid levels and risk of new‐onset atrial fibrillation: A systematic review and dose‐response meta‐analysis

**DOI:** 10.1002/clc.23430

**Published:** 2020-07-28

**Authors:** Yisong Yao, Feng Liu, Yangyang Wang, Zengzhang Liu

**Affiliations:** ^1^ Department of Cardiology The Second Affiliated hospital, Chongqing Medical University Chongqing China; ^2^ Department of Neurosurgery The Second Affiliated hospital, Chongqing Medical University Chongqing China

**Keywords:** atrial fibrillation, high‐density lipoprotein cholesterol, low‐density lipoprotein cholesterol, total cholesterol, triglyceride

## Abstract

Lipid levels are closely associated with health, but whether lipid levels are associated with atrial fibrillation (AF) remains controversial. We thought that blood lipid levels may influence new‐onset AF. Here, we used a meta‐analysis to examine the overall association between lipid levels and new‐onset AF. PubMed and EMBASE databases were searched up to 20 December 2019. We conducted a systematic review and quantitative meta‐analysis of prospective studies to clarify the association between lipid levels and the risk of new‐onset AF. Sixteen articles with data on 4 032 638 participants and 42 825 cases of AF were included in this meta‐analysis. The summary relative risk (RR) for a 1 mmol/L increment in total cholesterol (TC) was 0.95 (95% CI 0.93‐0.96, I^2^ = 74.6%, n = 13). Subgroup analyses showed that follow‐up time is a source of heterogeneity; for low‐density lipoprotein cholesterol (LDL‐C), RR was 0.95 (95% CI 0.92‐0.97, I^2^ = 71.5%, n = 10). Subgroup analyses indicated that adjusting for heart failure explains the source of heterogeneity; for high‐density lipoprotein cholesterol (HDL‐C), RR was 0.97 (95% CI 0.96‐0.99, I^2^ = 26.1%, n = 11); for triglycerides (TGs), RR was 1.00 (95% CI 0.96‐1.03, I^2^ = 81.1%, n = 8). Subgroup analysis showed that gender, age, follow‐up time, and adjustment for heart failure are sources of heterogeneity. Higher levels of TC, LDL‐C, and HDL‐C were associated with lower risk of new‐onset AF. TG levels were not associated with new‐onset AF in all subjects.

## 1 INTRODUCTION

Atrial fibrillation (AF) is the most common type of sustained cardiac arrhythmia and currently affects over 2.3 million American adults, a number that is expected to more than double in the next five decades.^1^ AF is associated with increased risk of heart failure, stroke, and death from cardiovascular disease. Major risk factors for AF include age, white race, European, obesity, hypertension, lack of physical activity, sedentary lifestyle, smoking, alcohol intake, diabetes mellitus, and obstructive sleep apnea. Many of these predictors are also risk factors for coronary heart disease (CHD). Hyperlipidemia is a major contributor to the development of atherosclerosis and CHD. Higher levels of low‐density lipoprotein cholesterol (LDL‐C) and lower levels of high‐density lipoprotein cholesterol (HDL‐C) have been consistently associated with increased risk of CHD. Lowering of LDL‐C and total cholesterol (TC) with statins reduces the risk of coronary events. However, a large randomized clinical trial (ALLHAT) showed no relationship between use of pravastatin and incidence of AF. These data are consistent with a meta‐analysis in which an analysis of randomized controlled trials showed no significant effect of statins on the incidence of AF.^2^

Since hyperlipidemia is a risk factor for other cardiac conditions, it seems likely that hyperlipidemia would also be a risk factor for AF. There is, however, a “cholesterol paradox” in AF^3^, ^4^ and the association between lipid levels and the risk of new‐onset AF is less clear. Many recent epidemiological studies have explored association between lipid levels and the risk of new‐onset AF, some studies showed no significant association,^5^, ^6^, ^7^, ^8^, ^9^, ^10^, ^11^ and some studies were associated with lower risk.^4^, ^12^, ^13^, ^14^, ^15^, ^16^, ^17^ Given the increasing prevalence of AF globally, establishing the association between lipid levels and new‐onset AF is of major public health importance. For these reasons, we conducted a systematic review and meta‐analysis of prospective studies exploring the link between lipid levels and risk of new‐onset AF in order to clarify the direction and strength of the association.

## 2 METHODS

We followed the preferred reporting protocol for systematic reviews and meta‐analyses set out in the PRISMA Statement^18^ (CRD42020162579).

### 2.1 Literature search

We conducted a comprehensive literature search in the PubMed and EMBASE databases to evaluate the association between lipid levels and incidence of AF using the following search terms: “AF,” “TC,” “LDL‐C,” “HDL‐C,” “triglyceride (TG),” “AF”, “TC”, “LDL”, “HDL” and “TG”.

### 2.2 Inclusion and exclusion criteria

We used the following inclusion criteria: (a) the paper evaluated associations of lipid levels with the incidence of AF, (b) the paper provided adjusted relative risk estimates (hazard ratio, risk ratio) with 95% confidence intervals (CIs), and for dose‐response analyses, provided a quantitative measure of exposure and the total number of cases and person‐years or continuous risk estimate, and (c) when multiple publications were available from the same study, we used the study with the largest number of cases. We excluded the literature using the following criteria: (a) when multiple publications were available from the same study, we used the study with the largest number of cases; (b) when the publication was a meeting abstract with insufficient data available online; and (c) when the publication described a meta‐analysis, review, case‐control study, or cross‐sectional study. We reviewed all relevant studies and identified 64 published articles that discussed the association between blood lipid levels and incidence of AF; 16 articles met our inclusion criteria.

### 2.3 Data abstraction

Data abstraction was carried out independently by two authors (Yisong Yao and Yangyang Wang) and disagreements were resolved through discussion. The following information was abstracted: first author's last name, publication year, country where the study was conducted, study period, sample size, number of cases/controls, exposure variables, exposure levels, adjusted relative risk estimates (hazard ratio, risk ratio), and 95% CIs for the highest vs the lowest level of the exposure variable in the publication.

### 2.4 Quality assessment

Quality assessment of the publications was carried out using the Newcastle‐Ottawa Quality Assessment Scale (NOS), which has a nine‐point scale (four for quality of selection, two for comparability, and three for quality of outcome and adequacy of follow‐up). The literature was divided into high quality (score ≥8) and low quality (score <8) **(**Supplementary Table 1 **)**.

### 2.5 Statistical analysis

All of our results were analyzed using the Stata 14.0 software. We calculated summary relative risks (RRs) and 95% CIs for 1 mmol/L increments in TC, LDL‐C, HDL‐C, and TGs, using a random effects model or a fixed effects model, which takes into account heterogeneity between studies. RRs were pooled using a fixed effect model if I^2^ was lower than 50%, otherwise the random effect model was used. Publication bias was estimated using Egger's test and Begg's test. Subgroup analyses were completed using the characteristics of studies to find sources of heterogeneity. To verify the stability of our results, a sensitivity analysis was performed, in which one study at a time was removed and the remaining studies analyzed to evaluate whether the result could have been affected markedly by a single study.

We used the method described by Orsini et al to analyze dose‐responses of lipid levels and calculated study‐specific slopes (linear trends) and 95% CIs from the natural logarithms of the reported RRs and CIs across categories of each lipid level.^19^ The mean lipid level in each category was assigned to the corresponding RR for each study and, for studies that reported exposures in ranges, we calculated the average of the upper and the lower cutoff points and used this as a midpoint. When the lowest or highest category was open‐ended or had an extreme range, we used the width of the adjacent interval to calculate an upper or lower cutoff value. For studies that reported continuous risk estimates per 10 mg/dL or per 1.17 mmol/L, these risk estimates were converted to a risk estimate per 1 mmol/L lipid by taking the natural logarithm (ln) of the RR (95% CI), dividing the ln (RR, 95% CI) by the increment reported, multiplying by 3.9 and then back transforming to a nonlogarithmic scale before inclusion in the meta‐analysis. A potential nonlinear dose‐response relationship of TC, LDL‐C, HDL‐C, and TGs with risk of new‐onset AF was examined using fractional polynomial models.^19^ We determined the best‐fitting second order fractional polynomial regression model, defined as the one with the lowest deviance. A likelihood ratio test was used to assess the difference between nonlinear and linear models to test for nonlinearity.^19^ Since the nonlinear dose‐response analysis requires that data are reported for at least three of the categories TC, LDL‐C, HDL‐C, and TGs, studies that reported only a continuous risk estimate and not categorical data were excluded from the analysis.

## 3 RESULTS

**FIGURE 1:**
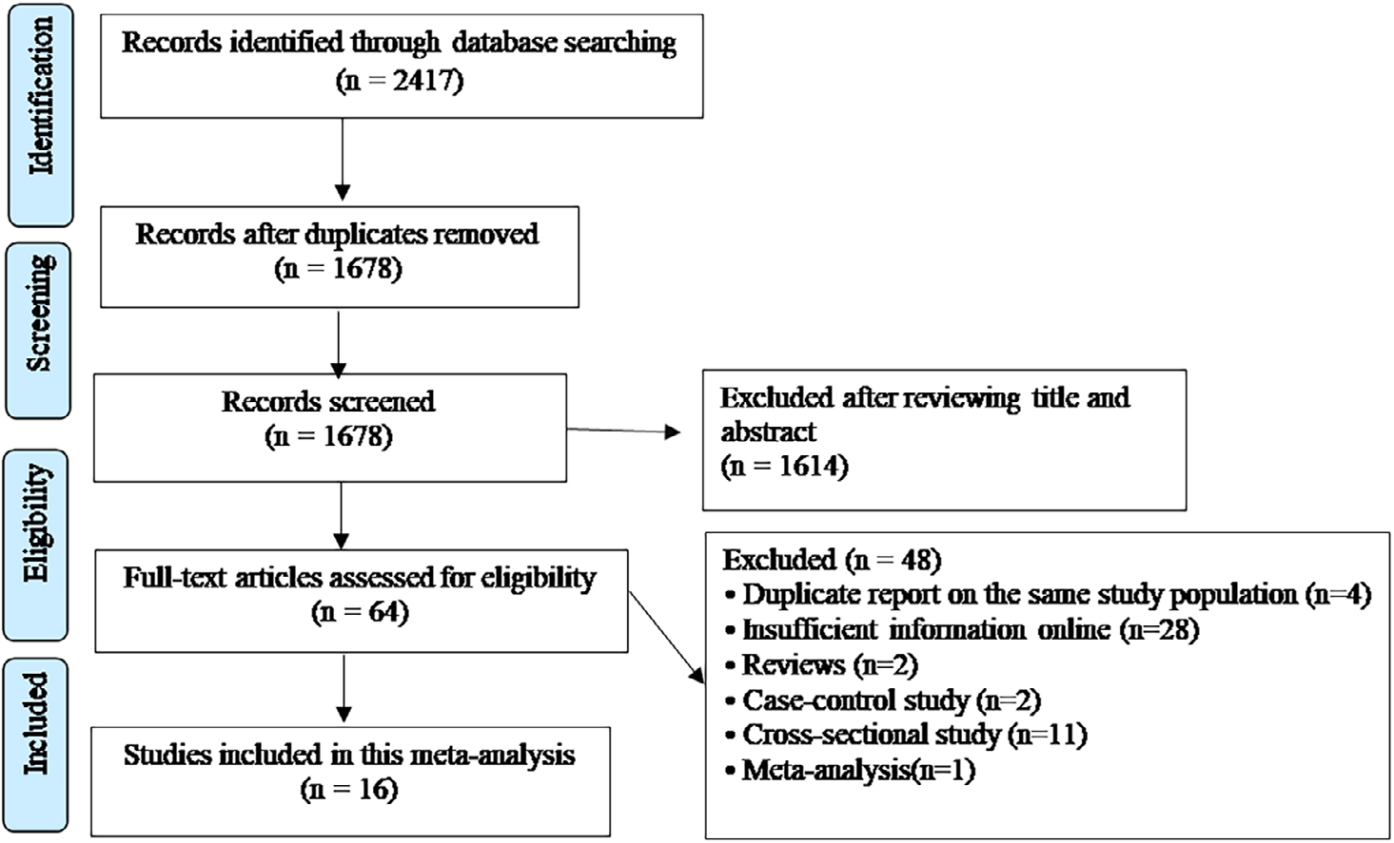
The flowchart of selecting eligible studies

The processes for retrieving and filtering articles, together with outcomes, are shown in Figure 1. After removal of duplicates, a preliminary screen of our search terms in the PubMed and EMBASE databases identified 1678 citations. We were able to exclude 1614 citations that did meet our criteria by reading the title or abstract, leaving 64 articles. After reading the full texts of these 64 articles, 48 were excluded **(Supplementary Table** **2** **)**. Four articles were excluded because they were a duplicate report on the same cohort population and 28 articles were excluded because insufficient information was available online. Two reviews, one meta‐analysis, two case‐control studies, and 11 cross‐sectional studies were also excluded, leaving 16 articles^4^, ^5^, ^6^, ^7^, ^8^, ^9^, ^10^, ^11^, ^12^, ^13^, ^14^, ^15^, ^16^, ^20^, ^21^, ^22^ for inclusion in our systematic review (Figure 1).

The characteristics of these 16 publications are shown in Table 1. The age range or mean age for each study is provided; the lowest and highest ages of participants across all studies were 18 and 96 years, respectively. The duration of follow‐up was between 3.28 and 40 years. One study included only men; fifteen cohort studies included both men and women. Four studies were carried out in Asia and 12 studies were carried out in the United States or Europe.

**TABLE 1:** Characteristics of 16 studies of lipid levels and the risk of new‐onset AF

Author	Year	Location	Sample size	Mean age, y	Mean follow‐up, y	No. of cases	Adjustment factors
Hallström et al^20^	2019	Sweden	(M + W) 36 258	35.6	9.7	947	Adjusted for time‐updated age, sex, education, born in Sweden, time‐updated diabetes duration and baseline comorbidities, time‐updated variables of smoking, HbA1c, SBP, and BMI.
Mourtzinis et al^12^	2018	Sweden	(M + W) 51 020 M 28211 W22,809	64	3.5	2389	Adjusted for age and SBP, DM, heart failure, ischemic heart disease, cerebrovascular disease, heart valvular disease, chronic kidney disease, thyroid disorder, chronic obstructive pulmonary disease, obstructive sleep apnea, alcohol abuse, antihypertensive medication, lipid‐lowering medication, antidiabetic medication, smoking habits, place of birth, education level, and BMI.
Li et al^13^	2017	China	(M + W) 88 785	50.83	7.12	328	Adjusted for sex, age, education, income, smoking, alcohol use, SBP, DBP, BMI, height, physical activity, hs‐CRP, SUA, DM, antihypertensive drugs, snoring.
Kokubo et al^5^	2017	Japan	(M + W) 6898	55.7	13.9	311	Basic risk factors and age‐ and sex‐adjusted hazard ratios for incident atrial fibrillation.
Magnussen et al^14^	2017	Europe	(M + W) 79 793 W41,226 M38,567	49.2 50	12.4	4261 1796 2465	Adjusted for sex, BMI, SBP, daily smoking, diabetes mellitus, and antihypertensive medication.
Sciacqua et al^21^	2015	Italy	(M + W) 3549	60.7	3.44	546	Adjusted for age, gender, glucose, LDL‐cholesterol, smoking, BMI, and SBP.
Eryd et al^22^	2014	Swedish	(M + W) 4846	56.8	15.3	353	Adjusted for age, gender, risk factors of AF.
Watanabe et al^15^	2012	Japan	(M + W) 28 449	59	4.5	265	adjusted for sex, age, BMI, systolic and diastolic blood pressure, and fasting blood sugar.
Nyrnes et al^8^	2012	Norway	(M + W) 22 815	46	11.1	822	Multivariable‐adjusted.
Faye L et al^10^	2013	America	(M + W) 13 969	54	18.7	1433	Adjusted for age, sex and race, study site, education, income, height, smoking status, drinking status, BMI, SBP, DBP, use of antihypertensive medication, diabetes, prevalent stroke, prevalent heart failure, and prevalent coronary heart disease
Rosengren et al^11^	2009	Sweden	M 6903	51.5	34.3	1253	Adjusted for age
Alonso et al^7^	2014	America	(M + W) 7142	61	9.6	480	Adjusted for age, sex, and race or ethnicity, study site (only in MESA), education, height, BMI, smoking status, alcohol drinking, physical activity, systolic and diastolic blood pressure, use of antihypertensive medication, diabetes, C‐reactive protein, and loge (NT‐proBNP) (in MESA) or loge(BNP) (in the FHS),incident myocardial infarction and incident heart failure as time‐dependent covariates
Lee et al^4^	2019	Korea	(M + W) 3 660 385	43.4	5.38	27 581	Adjusted for age, sex, smoking, alcohol use, regular exercise, income status, presence of hypertension, DM, baseline body mass index, glucose, SBP, and estimated glomerular filtration rate
Knuiman et al^6^	2013	Australia	(M + W) 4267	52	15	343	Adjusted for sex, age, height, hypertension treatment and BMI terms.
Psaty BM et al^16^	1997	America	(M + W) 4844	> = 65	3.28	304	DBP, weight, history of high blood pressure, sex, serum creatinine, history of congestive heart failure, history of cerebrovascular disease, diabetes, estrogen use, fibrinogen, self‐assessed health status, potassium, current smoking, ACE inhibitors, vasodilators, calcium‐channel blockers, ankle‐arm index, major ECG abnormalities, left ventricular hypertrophy by ECG, left ventricular ejection fraction, left ventricular systolic wall motion abnormalities, aortic root dimension, maximum intimal‐medial thickness of the common carotid artery, maximum intimal‐medial thickness of the internal carotid, forced vital capacity, and HDL cholesterol level.
Alonso et al (2013)‐CHARGE‐AGES^9^	2006	Europe	(M + W) 4469	76	40	408	Adjusted for age and sex
Alonso et al (2013)‐CHARGE‐RS^9^	1999	Europe	(M + W) 3203	72	10	177	Adjusted for age and sex
Alonso et al (2013)‐CHARGE‐CHS AA^9^	1999	America	(M + W) 719	73	10	64	Adjusted for age and sex
Alvaro CHARGE‐CHS‐white)	1999	America	(M + W)4324	73	10	560	Adjusted for age and sex

Abbreviations: AF, atrial fibrillation; BMI, body mass index; DBP, diastolic blood pressure; DM, diabetes mellitus; ECG, electrocardiogram; M, men; M + W, men+women; SBP, systolic blood pressure; SUA, serum uric acid; W, women.

### 3.1 Total cholesterol

**FIGURE 2:**
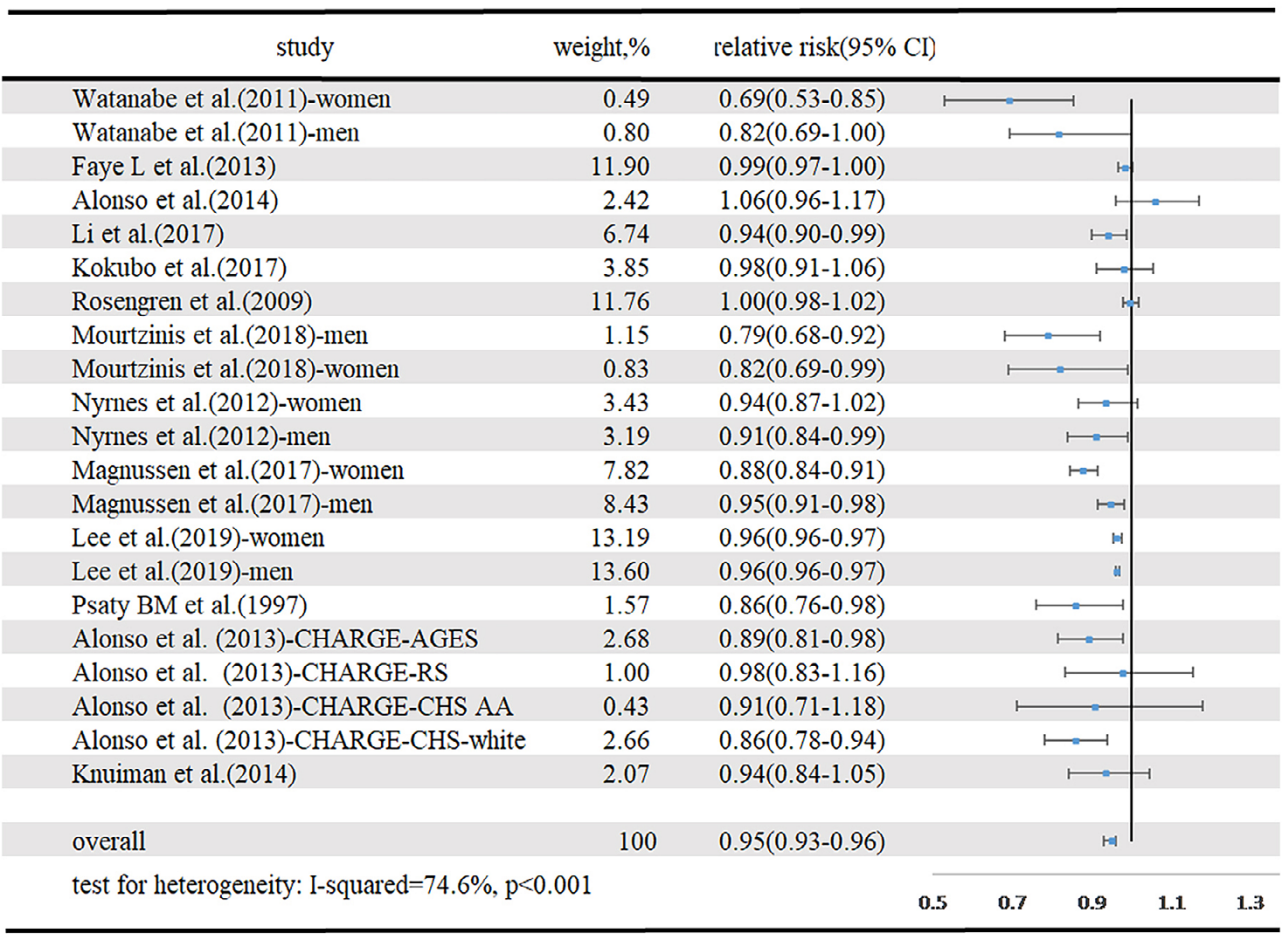
Forest plot for TC and risk of new‐onset AF, per 1 mmol/L TC increase. AF, atrial fibrillation; CI, confidence interval; TC, total cholesterol

Thirteen articles, with 40 979 cases of new‐onset AF among 3 987 985 participants^4^, ^5^, ^6^, ^7^, ^8^, ^9^, ^10^, ^11^, ^12^, ^13^, ^14^, ^15^, ^16^ were included in our analysis of the dose‐response between TC and the incidence of new‐onset AF. The summary RR for a 1 mmol/L increment in TC was 0.95 (95% CI 0.93‐0.96, I^2^ = 74.6%, *P* < .001) (Figure 2). There was some indication of publication bias (Egger's test, *P* = .025; Begg's test, *P* = .057). Six studies were included in the nonlinear dose‐response analysis.^2^, ^3^, ^5^, ^8^, ^9^, ^11^ The dose‐response curve, which is U‐shaped, is shown in **S**u**pplementary Figure** **1**. TC concentrations in the range 232 to 238 mg/dL (6.009‐6.164 mmol/L) were associated with lower risk of new‐onset AF.

### 3.2 Low‐density lipoprotein cholesterol

**FIGURE 3:**
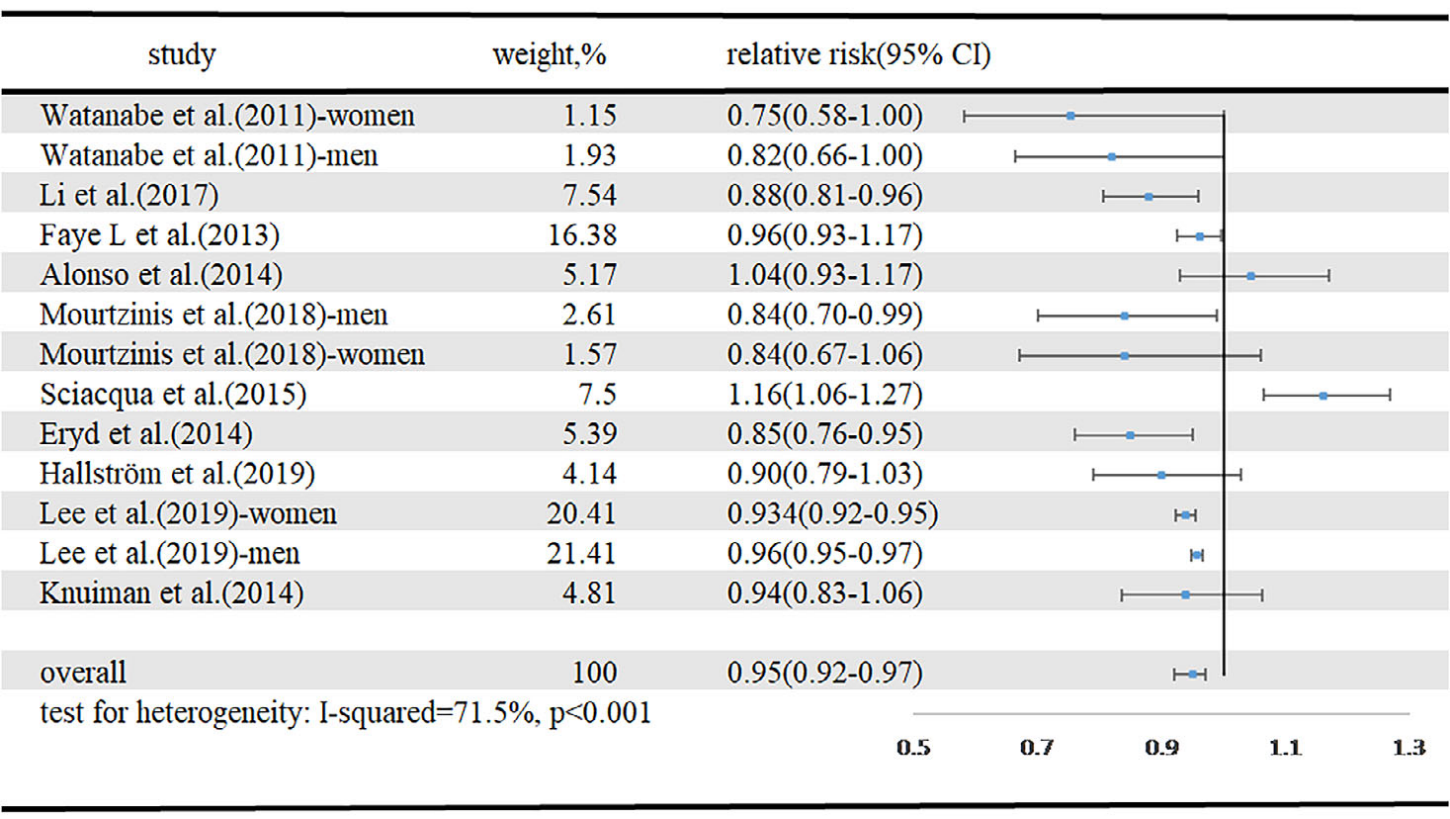
Forest plot for LDL‐C and risk of new‐onset AF, per 1 mmol/L LDL‐C increase. AF, atrial fibrillation; CI, confidence interval; LDL‐C, low‐density lipoprotein cholesterol

Ten articles with 34 665 cases of new‐onset AF among 3 898 670 participants^4^, ^6^, ^7^, ^10^, ^12^, ^13^, ^15^, ^20^, ^21^, ^22^ were included in our analysis of the dose‐response between LDL‐C and the incidence of new‐onset AF. The summary RR for a 1 mmol/L increment in LDL‐C was 0.95 (95% CI 0.92‐0.97, I^2^ = 71.5%, *P* < .001) (Figure 3). There was no indication of publication bias (Egger's test, *P* = .393; Begg's test, *P* = .300). Four studies were included in the nonlinear dose‐response analysis.^4^, ^7^, ^10^, ^13^ The dose‐response curve, which has a reversed spoon shape is shown in Supplementary Figure 2. LDL‐C concentrations in the range 133 to 149 mg/dL (3.445‐3.859 mmol/L) were associated with lower risk of new‐onset AF.

### 3.3 High‐density lipoprotein cholesterol

**FIGURE 4:**
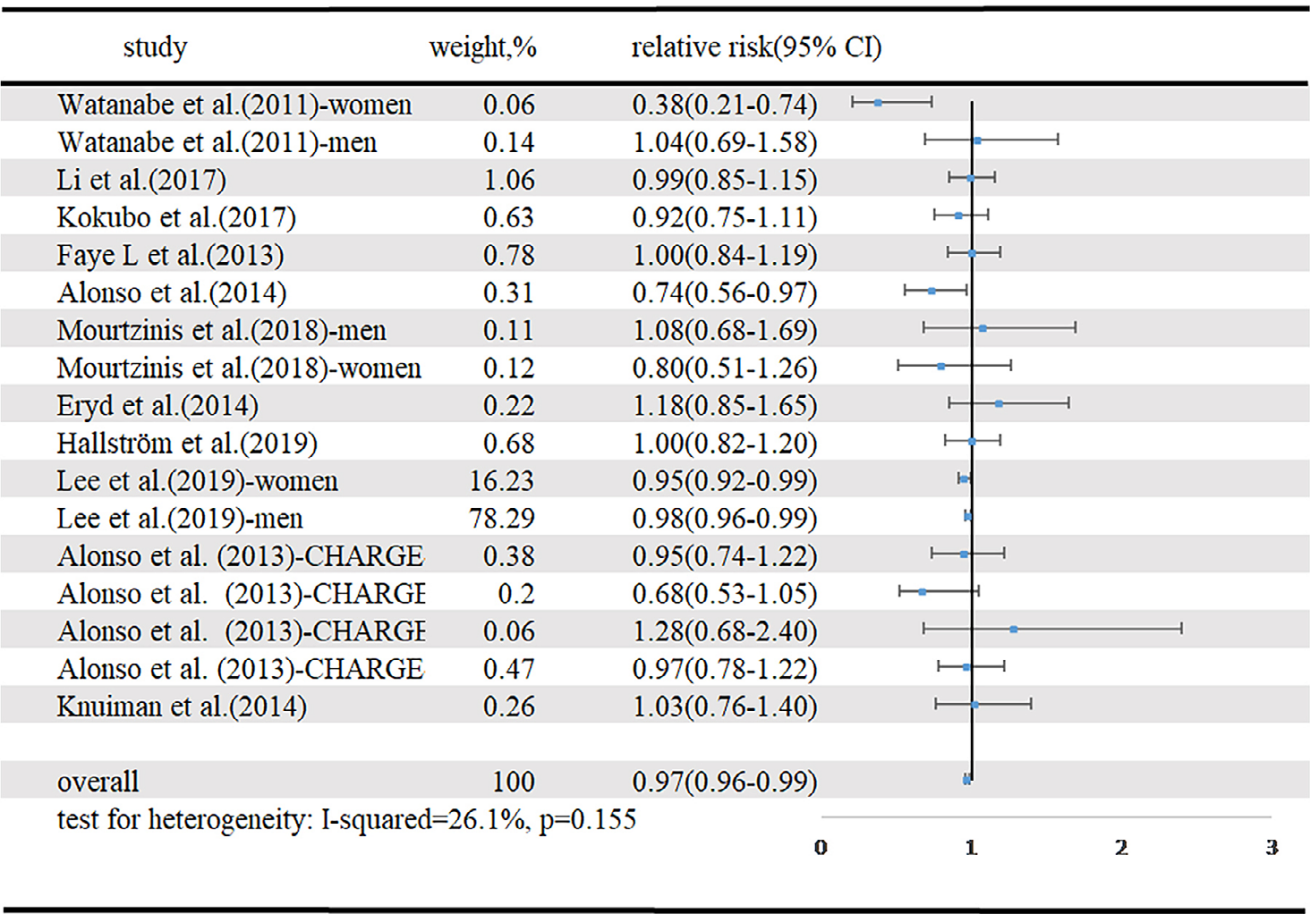
Forest plot for HDL‐C and risk of new‐onset AF, per 1 mmol/L HDL‐C increase. AF, atrial fibrillation; CI, confidence interval; HDL‐C, high‐density lipoprotein cholesterol

Eleven articles with 35 639 cases of new‐onset AF among 3 914 734 participants,^4^, ^5^, ^6^, ^7^, ^9^, ^10^, ^12^, ^13^, ^15^, ^20^, ^22^ were included in our analysis of the dose‐response between HDL‐C and the incidence of new‐onset AF. The summary RR for a 1 mmol/L increment in HDL‐C was 0.97 (95% CI 0.96‐0.99, I^2^ = 26.1%, *P* < .401) (Figure 4). There was no indication of publication bias (Egger's test, *P* = .249; Begg's test, *P* = .773). Five studies were included in the nonlinear dose‐response analysis.^2^, ^3^, ^5^, ^8^, ^11^ The dose‐response curve, which is linear, is shown in Supplementary Figure 3. HDL‐C concentrations in the range 31 to 80 mg/dL (0.803‐2.072 mmol/L) were linearly and negatively correlated with new‐onset AF.

### 3.4 Triglycerides

Eight articles with 33 996 cases of new‐onset AF among 3 869 363 participants^4^, ^5^, ^7^, ^9^, ^10^, ^12^, ^13^, ^15^ were included in our analysis of the dose‐response between TGs and the incidence of new‐onset AF. The summary RR for a 1 mmol/L increment in TGs was 1.00 (95% CI 0.96‐1.03, I^2^ = 81.1%, *P* < 0.001) (Supplementary Figure 4). There was no indication of publication bias (Egger's test, *P* = .551; Begg's test, *P* = .443).

### 3.5 Heterogeneity test

Significant heterogeneity was found in our meta‐analysis. TC, LDL‐C, and TGs were used for subgroup analyses. Subgroup analyses may be the source of heterogeneity.

In the case of TC, two factors need to be taken into account. First, age should be taken into account since this has a significant impact on AF; heterogeneity was noticeably different among the age subgroups in our study. Greater heterogeneity was found in the subgroups *age < 50 years* (I^2^ = 78.80%, *P* < .001), *age < 60 years* (I^2^ = 67.50%, *P* = .005), and *age < 70 years* (I^2^ = 84.50%, *P* < .001); the subgroup *age > 70 years* showed less heterogeneity (*P* = .71, I^2^ = 0.0%). Second, follow‐up time may also influence heterogeneity. Greater heterogeneity (*P* < .001, I^2^ = 82.30%) was found in the subgroup *follow‐up time > 10 years*, and less heterogeneity was found in the subgroups *follow‐up time < 5 years* (*P* = .615, I^2^ = 0%) and *follow‐up time 5‐10 years* (*P* = .069, I^2^ = 46.7%) (Supplementary Figure 5).

In the case of LDL‐C, two factors must also be taken into account. First, gender should be taken into account since this has an obvious impact on AF. Less heterogeneity (*P* = .19, I^2^ = 40.50%) was found in the subgroup *women*; higher heterogeneity was found in the subgroups *men* (*P* < .001, I^2^ = 79.50%) and *men and women* (*P* = .11, I^2^ = 54.2%). Second, heart failure should also be taken into account since this has a significant impact on AF. Less heterogeneity (*P* = .10, I^2^ = 43.80%) was found in the subgroup that was adjusted for heart failure and higher heterogeneity (*P* < .001, I^2^ = 84.10%) was found in the subgroup that was not adjusted for heart failure (Supplementary Figure 6).

In the case of TGs, four factors should be taken into account. First, gender should be taken into account since this has a significant impact on AF. Less heterogeneity was found in the subgroups *women* (*P* = .249, I^2^ = 28%) and *men* (*P* = .557, I^2^ = 0%); higher heterogeneity was found in the subgroup *men and women* (*P* = .015, I^2^ = 59.8%,). The summary RR was 0.93 (95% CI 0.92‐0.95, I^2^ = 0, n = 3) per 1 mmol/L increment in men. Second, age has a significant impact on AF. Less heterogeneity was found in the subgroup *age 51 to 60 years* (*P* = .807, I^2^ = 0%,) and the subgroup *age ≥ 70 years* (*P* = .23, I^2^ = 30.4%). Third, follow‐up time has a significant impact on AF. Less heterogeneity was found in the subgroup *follow‐up time < 5 years* (*P* = .364 I^2^ = 7.5%) and the subgroup *follow‐up time > 10 years* (*P* = .372, I^2^ = 0%). Fourth, heart failure has a significant impact on AF. Less heterogeneity was found in the subgroup that was adjusted for heart failure (*P* = .806, I^2^ = 0%) and higher heterogeneity was found in the subgroup that was not adjusted for heart failure (*P* < .001, I^2^ = 76.8%) (Supplementary Figure 7).

Finally, we performed sensitivity analysis. Since all the results were statistically significant, we had achieved a relativity stable outcome.

## 4 DISCUSSION

### 4.1 Major outcomes

To our knowledge, this is the first meta‐analysis of lipid levels and the risk of new‐onset AF. There was a 5% decrease in RR per 1 mmol/L increase in TC, a 5% decrease in RR per 1 mmol/L increase in LDL‐C, and a 3% decreases in RR per 1 mmol/L increase in HDL‐C. No association was observed between TGs and new‐onset AF in all subjects, but subgroup analysis found that the summary RR was 0.93 (95% CI 0.92‐0.95, I^2^ = 0, n = 3) per 1 mmol/L increment in men. The dose‐response curve between TC and AF was U‐shaped and that between LDL‐C and AF had a reversed spoon shape; a linear association was observed between HDL‐C and AF.

### 4.2 Possible biological mechanisms

Several mechanisms may explain the negative correlation between blood lipids and new‐onset AF.

First, cholesterol is an essential component of the plasma membrane and there are numerous reports linking changes in membrane cholesterol to alterations in the functional properties of the membrane. These include changes in the functioning of ion channels, membrane‐associated enzymes and receptors, and even intracellular trafficking. Ion channels, which allow the flow of ions down their electrochemical gradient across the membrane, are key to both producing and regulating action potentials and there is much functional evidence that cholesterol regulates cardiac ion currents. The mechanisms underlying the effects of cholesterol on ion channels are obviously complex and are not yet fully understood.^23^, ^24^, ^25^, ^26^, ^27^, ^28^, ^29^, ^30^ Antonius et al found that dyscholesterolemia alters the lipid content of cardiac myocytes,^31^ which affects the properties of the ion channels underlying sodium and L‐type calcium currents, and results in a decrease of upstroke velocity and increased duration of the action potential and QT interval in the ECG. These electrophysiological changes resulted in reduced inducibility of lethal arrhythmias caused by acute myocardial infarction, an effect that appears to be similar to that seen with class III antiarrhythmic drugs. Further research is needed to fully elucidate the effects of cholesterol on ion channels and on membrane‐resident pumps, membrane receptors, and trafficking of membrane proteins, all of which may be affected by cholesterol.^32^

Second, the link between HDL‐C, LDL‐C, and new‐onset AF may be inflammation.^33^ There is plausible evidence linking inflammation to the initiation and perpetuation of AF,^34^ Inflammation is accompanied by an increase in cytokines, which leads to changes in metabolism of lipids and lipoproteins. LDL‐C and HDL‐C levels are known to be reduced in inflammation, which is related to the action of inflammatory cytokines; low levels of LDL‐C and HDL‐C can reflect levels of inflammation. Cholesterol is present in blood as lipoproteins, including HDL‐C, LDL‐C, and very low‐density lipoprotein cholesterol (VLDL‐C). Inflammation reduces the levels of HDL and LDL, resulting in decreasing serum levels of HDL‐C and LDL‐C. HDL metabolism is also tightly linked to reverse cholesterol transport (RCT), a process by which cholesterol is removed from peripheral cells and transported to the liver for metabolism and/or excretion. Several HDL‐associated proteins and a number of cell surface receptors play a key role in RCT and during infection and inflammation, there is a reduction in RCT that is attributable to multiple changes at each step in the pathway.^33^ TG levels are known to increase in inflammation and several cytokines are known to increase TG levels. Higher levels of TG reflect the level of inflammation within the host, but in our study, there was no association between TG and new‐onset AF.

Third, old age and hyperthyroidism are associated with low cholesterol levels and increased incidence of AF, which may be confounding factors or reflections of a hidden link behind cholesterol and AF. The incidence of AF increases with age, especially in older populations. Thyroid hormones upregulate LDL‐C receptors and increase cholesterol catabolism and excretion, resulting in a decrease in TC and LDL‐C, whereas HDL‐C is reduced or unaffected.^35^ Subclinical or clinical hyperthyroidism is strongly associated with the development of AF.

Fourth, Mora et al. found an inverse association between AF and cholesterol‐poor small LDL‐C particles and small VLDL particles, rather than with larger, cholesterol‐enriched particles.^17^ This suggests that there are likely mechanisms beyond direct cholesterol effects underlying the observed association between LDL‐C and new‐onset AF. Further research is needed to fully understand these mechanisms.

In our study, we found a nonlinear (U‐shaped) association between TC and new‐onset AF. We also found a nonlinear (reverse spoon‐shaped) association between concentrations of LDL‐C and the risk of new‐onset AF. Our study suggests that as a risk factor for new‐onset AF, neither “the higher, the better” nor “the lower, the better” is correct in terms of cholesterol concentration.^36^, ^37^, ^38^

### 4.3 Previous studies

Although most of the studies included here found that high cholesterol levels were associated with lower risk of new‐onset AF, the dose‐response curve was not clear and the best concentration range was unknown. Our quantitative meta‐analysis found a non‐linear relationship between both TC and LDL‐C and new‐onset AF. A negative relationship between HDL‐C and new‐onset AF was also found.

### 4.4 Limitations

In our meta‐analysis, although higher TC, LDL‐C, and HDL‐C were associated with lower risk of new‐onset AF, there are still some problems. First, our meta‐analysis showed an obvious heterogeneity between studies, which may affect the reliability of the results of the meta‐analysis and means that careful interpretation is needed. Second, there was publication bias with TC, which may affect the reliability of the results. Third, the heterogeneity was partially improved after subgroup analysis of follow‐up years, study quality, lipid‐lowering therapy, valvular atrial fibrillation, and diabetes. We believe that despite significant heterogeneity among studies, higher TC, LDL‐C, and HDL‐C were associated with lower risk of new‐onset AF. Our results provide an epidemiological basis for the underlying trials, but follow‐up studies on the relationship between new‐onset AF and cholesterol are needed.

## 5 CONCLUSIONS

Our meta‐analysis suggests that higher levels of TC, LDL‐C, and HDL‐C were associated with a lower risk of new‐onset AF and that TG levels were not associated with new‐onset AF across the entire study population. TC concentrations in the range 232 to 238 mg/dL (6.009‐6.164 mmol/L) were associated with lower risk of new‐onset AF, LDL‐C concentrations in the range 133 to 149 mg/dL (3.445‐3.859 mmol/L) were associated with lower risk of new‐onset AF, and HDL‐C concentrations in the range 31 to 80 mg/dL (0.803‐2.072 mmol/L) were linearly and negatively correlated with new‐onset AF.

## CONFLICT OF INTEREST

The authors declare no potential conflict of interest.

## AUTHOR CONTRIBUTIONS

Yisong Yao: Conceptualized the study; Yisong Yao, Yangyang Wang, and Feng Liu: Design of study; Yisong Yao, Feng Liu, and Yangyang Wang: Literature retrieval, study selection, data extraction, statistical analyses, interpretation of the data and drafting of the initial manuscript; Zengzhang Liu: Quality evaluation; Zengzhang Liu: Critical revision and comment for important intellectual content; all authors reviewed and approved the final manuscript.

## Supporting information


**Supplementary Figure 1** Dose‐response curve for relationship between TC and risk of new‐onset AF.
TC = total cholesterol, CI = confidence interval, AF = atrial fibrillation.

**Supplementary Figure 2** Dose‐response curve for relationship between LDL‐C and risk of new‐onset AF.
LDL‐C = low‐density lipoprotein cholesterol, CI = confidence interval, AF = atrial fibrillation.

**Supplementary Figure 3** Dose‐response curve for relationship between HDL‐C and risk of new‐onset AF.
HDL‐C = high‐density lipoprotein cholesterol, CI = confidence interval, AF = atrial fibrillation.

**Supplementary Figure 4** Forest plot for TGs and risk of new‐onset AF, per 1 mmol/L TGs increase.
TGs = triglycerides, CI = confidence interval, AF = atrial fibrillation.

**Supplementary Figure 5** Dose‐response relationship and subgroup analysis between TC and risk of new‐onset AF.
TC = total cholesterol, NOS = New Castle‐Ottawa Quality Assessment Scale,
CI = confidence interval, AF = atrial fibrillation.

**Supplementary Figure 6** Dose‐response relationship and subgroup analyses between LDL‐C and risk of new‐onset AF.
LDL‐C = low‐density lipoprotein cholesterol, NOS = New Castle‐Ottawa Quality Assessment Scale, CI = confidence interval, AF = atrial fibrillation.

**Supplementary Figure 7** Dose‐response relationship and subgroup analyses between TGs and risk of new‐onset AF.
TGs = triglycerides, NOS = New Castle‐Ottawa Quality Assessment Scale, CI = confidence interval, AF = atrial fibrillation.

**Supplementary Table 1** Results of quality assessment.
**Supplementary Table 2** Exclusion reasons
