# Supplementary figures and images for: Lipid levels and risk of new‐onset atrial fibrillation: A systematic review and dose‐response meta‐analysis
Source: Clin Cardiol. 2020 Jul 28;43(9):935–43. doi: 10.1002/clc.23430 (PMC7462197; doi:10.1002/clc.23430)

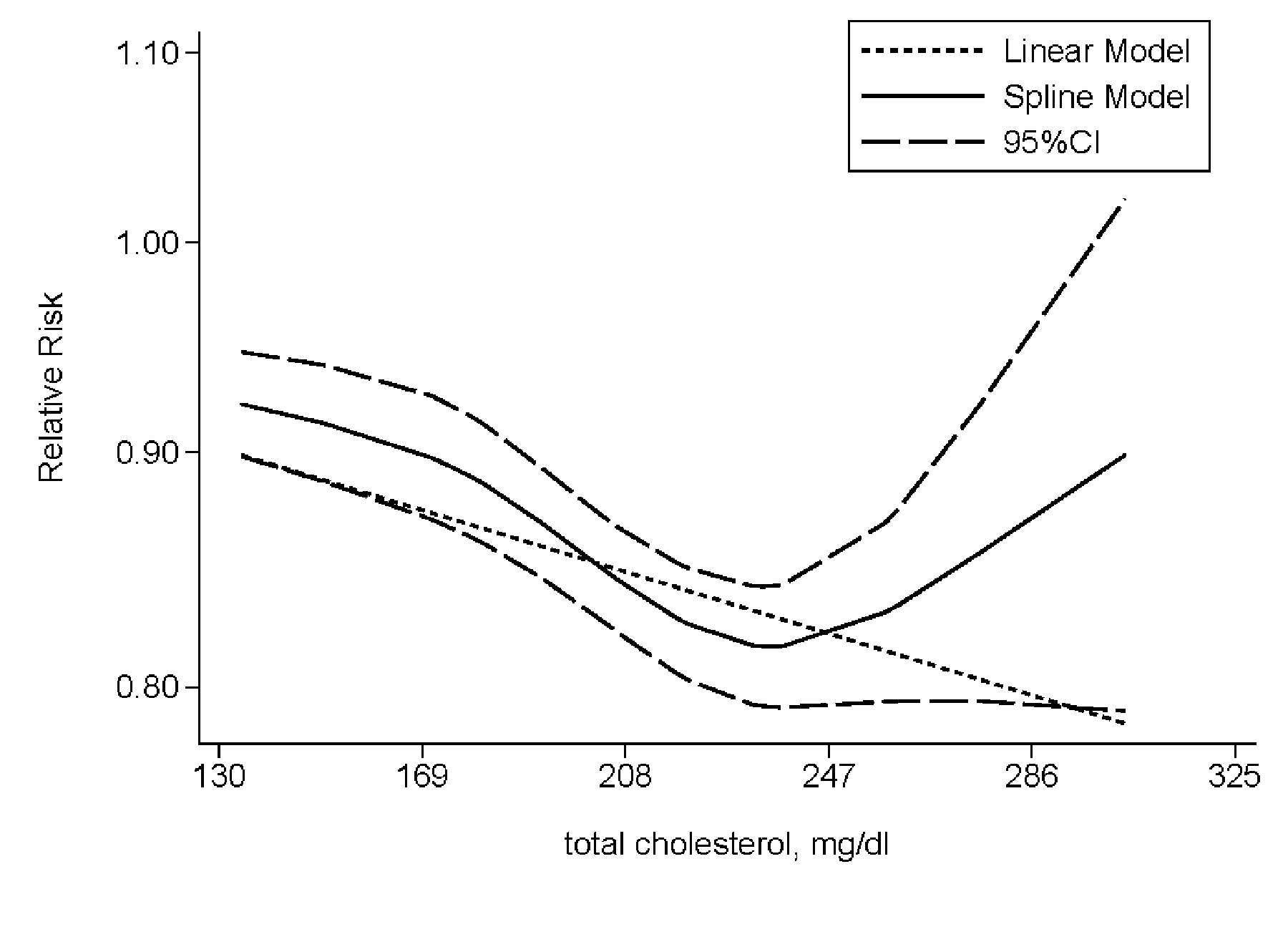

Supplement: Supplementary file 1 — Supplementary Figure 1 Dose‐response curve for relationship between TC and risk of new‐onset AF. TC = total cholesterol, CI = confidence interval, AF = atrial fibrillation. [file CLC-43-935-s001.tif]

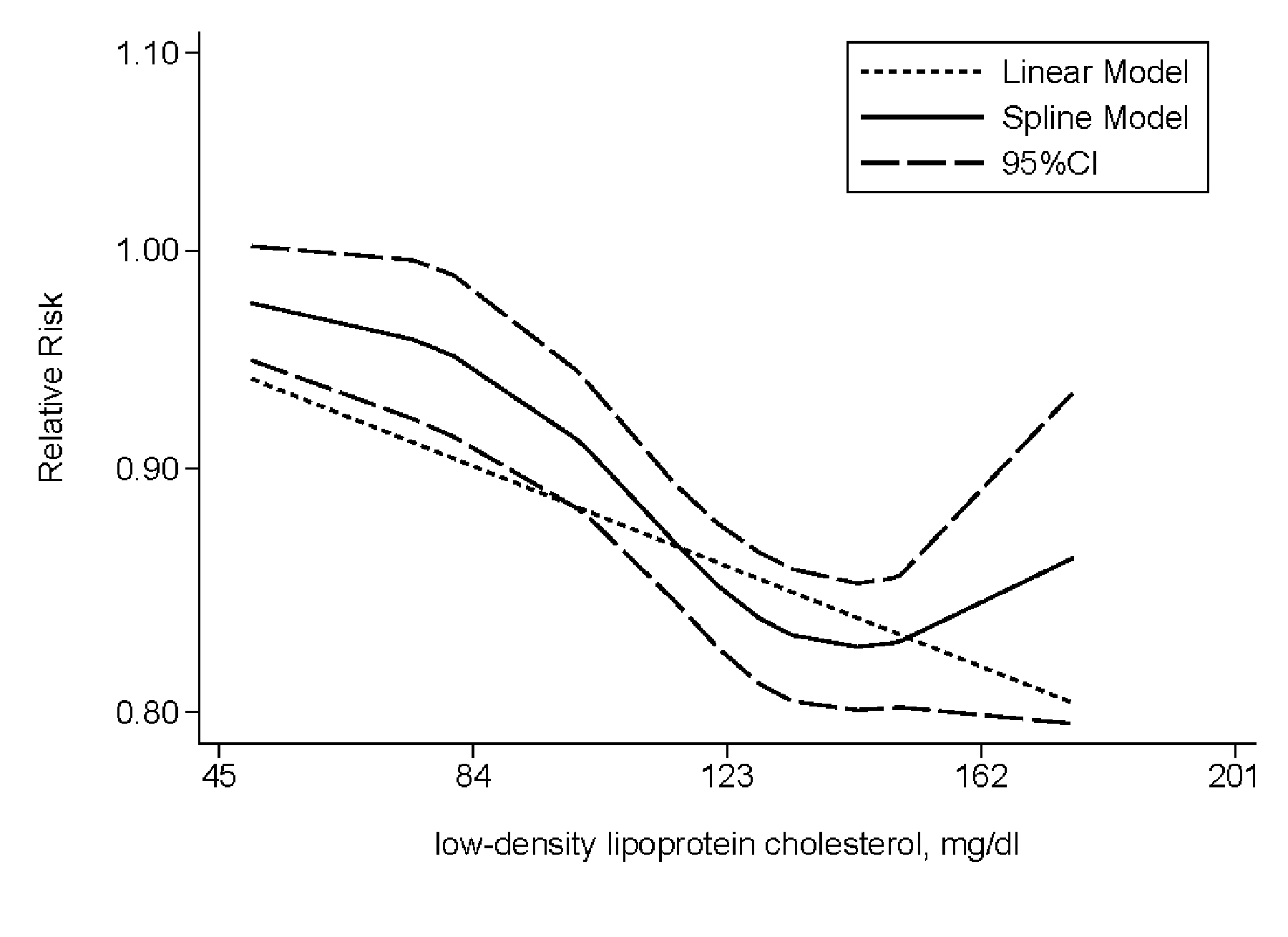

Supplement: Supplementary file 2 — Supplementary Figure 2 Dose‐response curve for relationship between LDL‐C and risk of new‐onset AF. LDL‐C = low‐density lipoprotein cholesterol, CI = confidence interval, AF = atrial fibrillation. [file CLC-43-935-s002.tif]

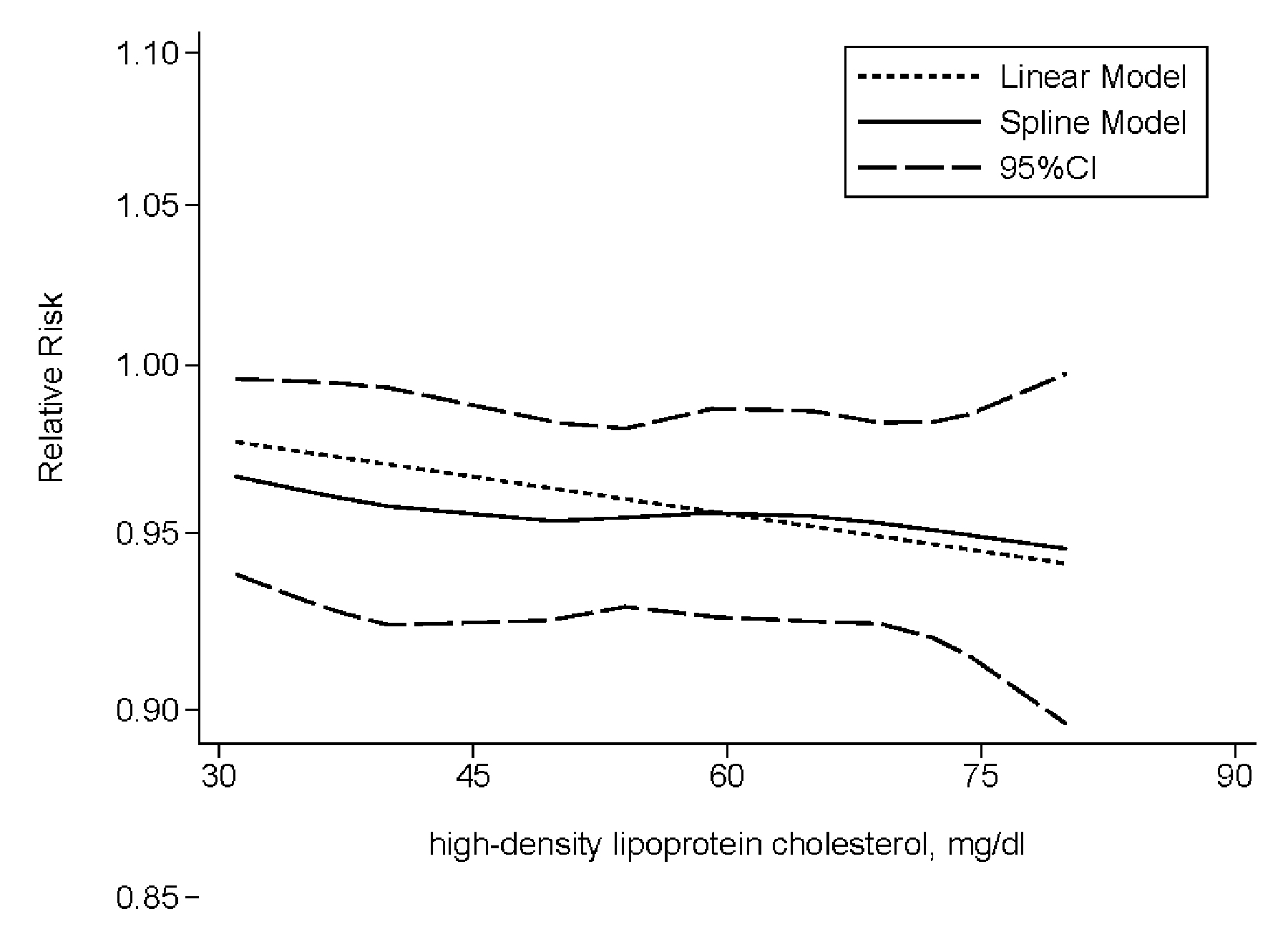

Supplement: Supplementary file 3 — Supplementary Figure 3 Dose‐response curve for relationship between HDL‐C and risk of new‐onset AF. HDL‐C = high‐density lipoprotein cholesterol, CI = confidence interval, AF = atrial fibrillation. [file CLC-43-935-s003.tif]

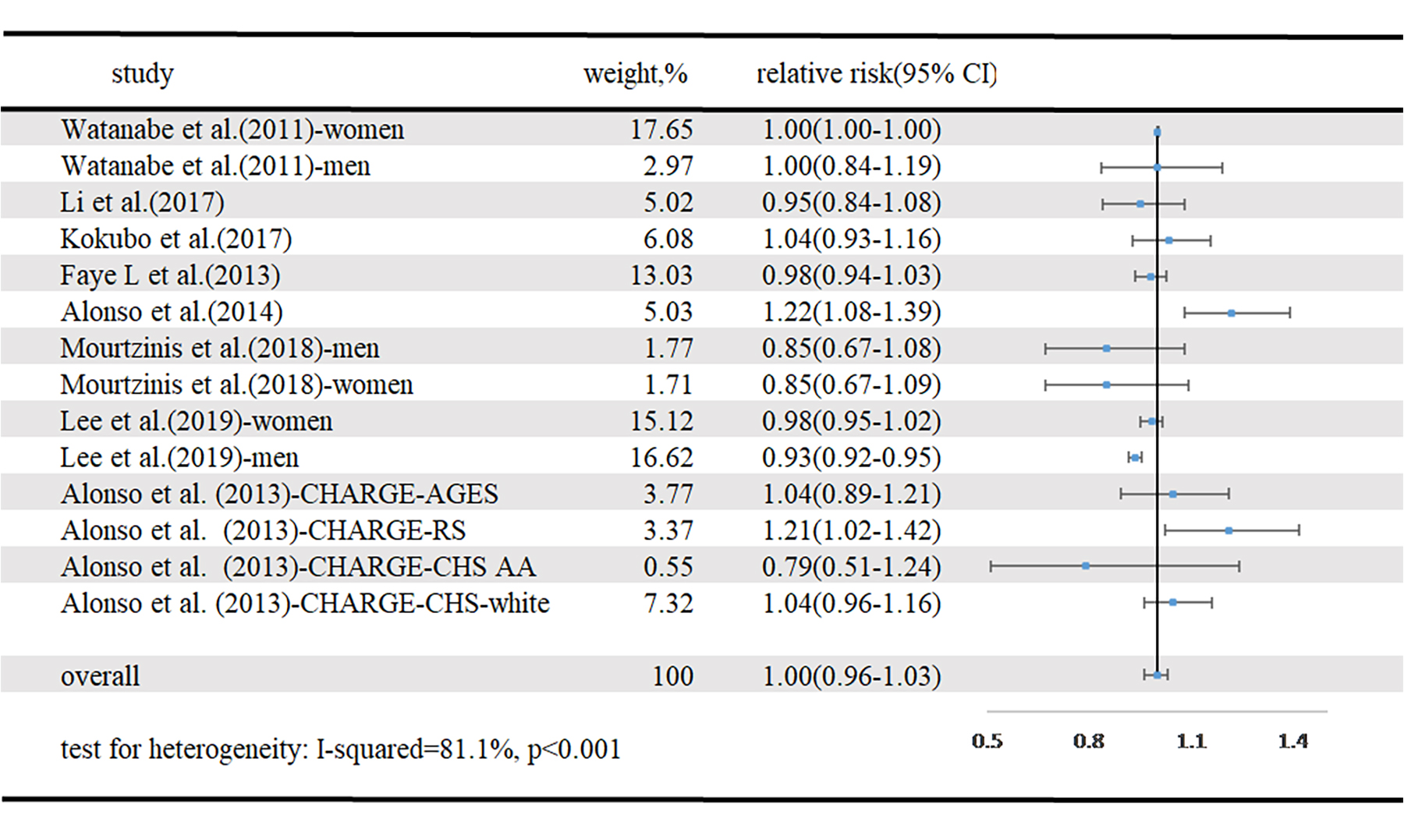

Supplement: Supplementary file 4 — Supplementary Figure 4 Forest plot for TGs and risk of new‐onset AF, per 1 mmol/L TGs increase. TGs = triglycerides, CI = confidence interval, AF = atrial fibrillation. [file CLC-43-935-s004.tif]

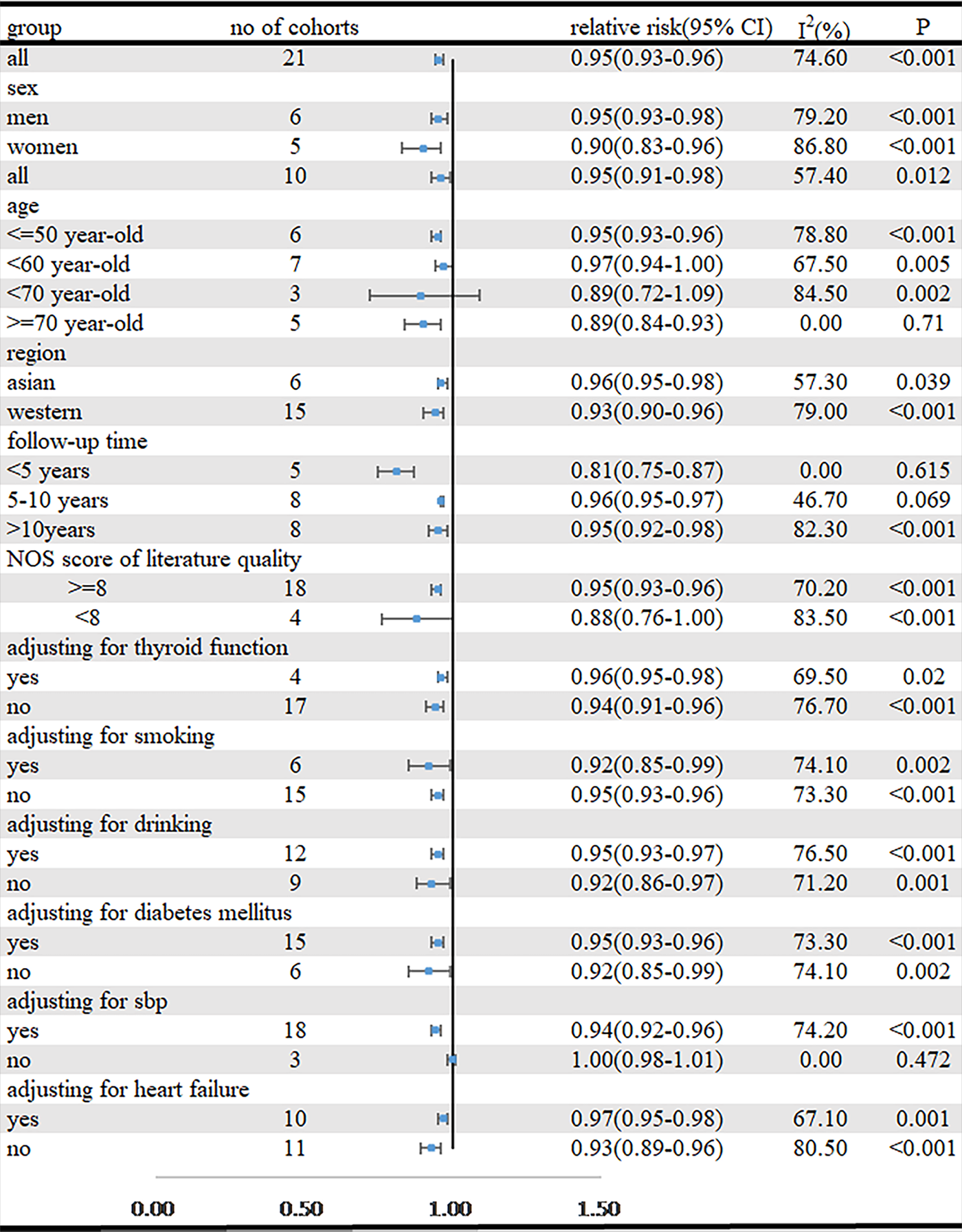

Supplement: Supplementary file 5 — Supplementary Figure 5 Dose‐response relationship and subgroup analysis between TC and risk of new‐onset AF. TC = total cholesterol, NOS = New Castle‐Ottawa Quality Assessment Scale, CI = confidence interval, AF = atrial fibrillation. [file CLC-43-935-s005.tif]

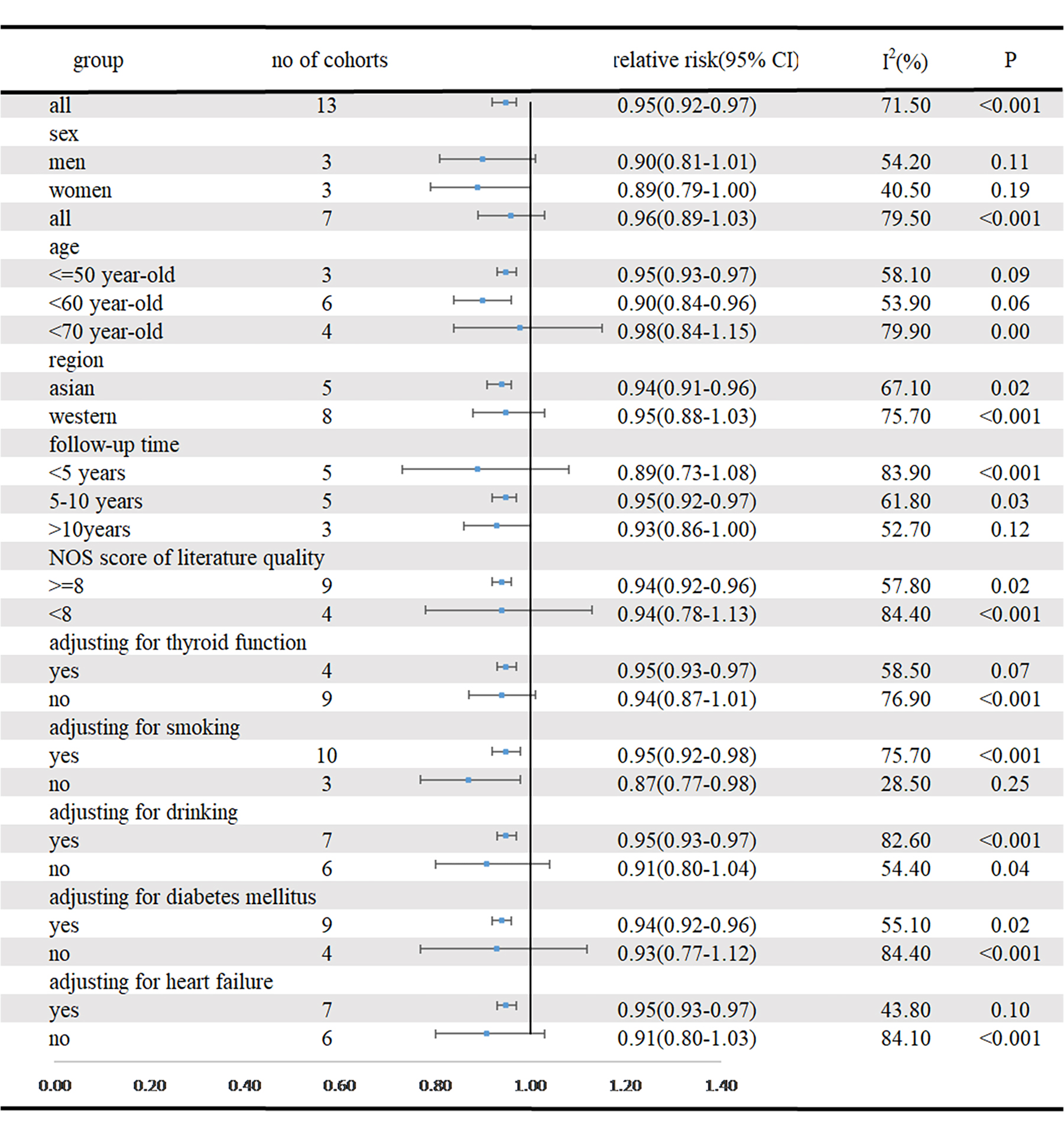

Supplement: Supplementary file 6 — Supplementary Figure 6 Dose‐response relationship and subgroup analyses between LDL‐C and risk of new‐onset AF. LDL‐C = low‐density lipoprotein cholesterol, NOS = New Castle‐Ottawa Quality Assessment Scale, CI = confidence interval, AF = atrial fibrillation. [file CLC-43-935-s006.tif]

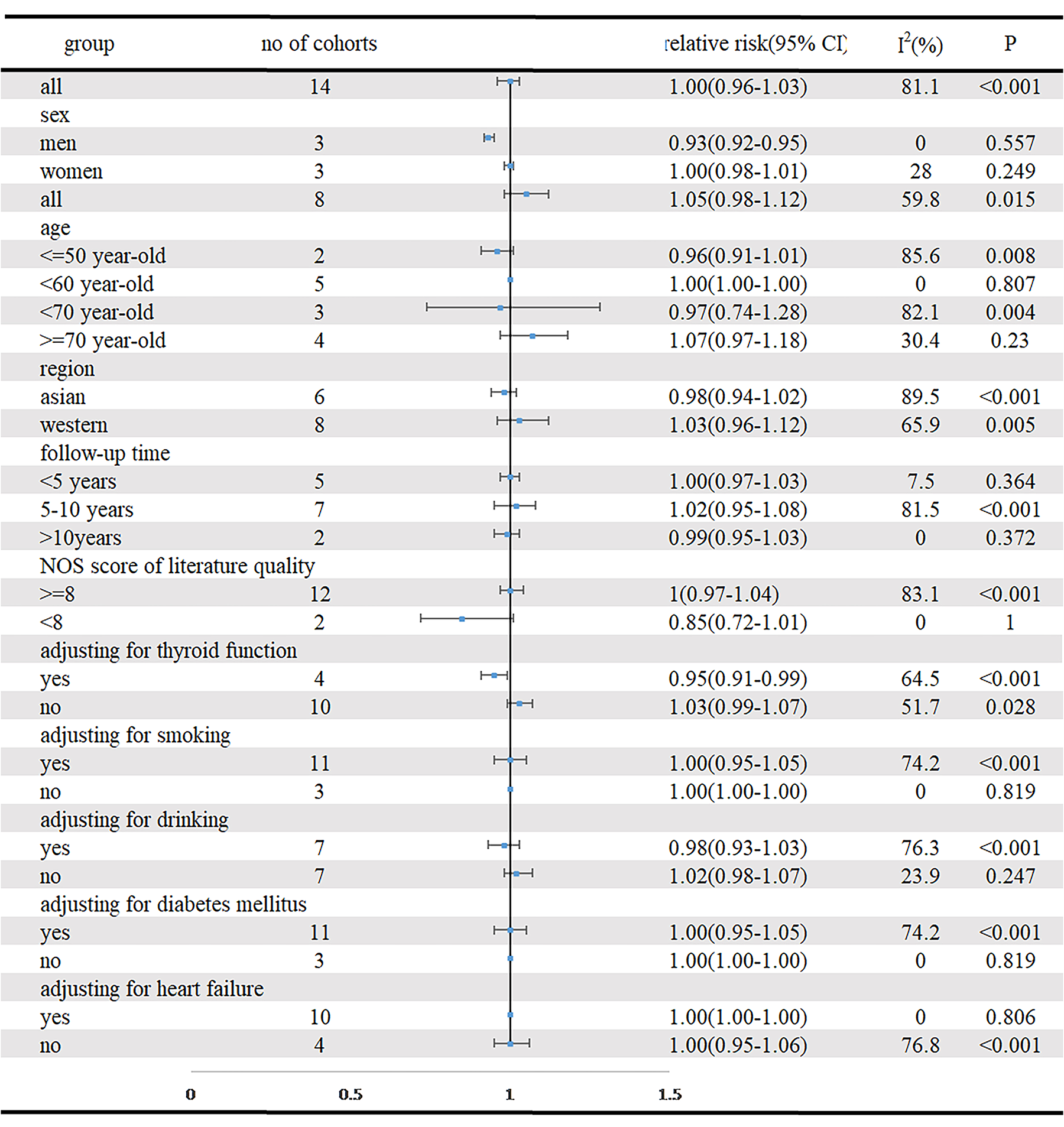

Supplement: Supplementary file 7 — Supplementary Figure 7 Dose‐response relationship and subgroup analyses between TGs and risk of new‐onset AF. TGs = triglycerides, NOS = New Castle‐Ottawa Quality Assessment Scale, CI = confidence interval, AF = atrial fibrillation. [file CLC-43-935-s007.tif]
