# Supplementary material for: Lipid levels and risk of new‐onset atrial fibrillation: A systematic review and dose‐response meta‐analysis
Source: Clin Cardiol. 2020 Jul 28;43(9):935–43. doi: 10.1002/clc.23430 (PMC7462197; doi:10.1002/clc.23430)
Supplement: Supplementary file 8 — Supplementary Table 1 Results of quality assessment. Supplementary Table 2 Exclusion reasons [file CLC-43-935-s008.docx]

**S**u**pplementary Table 1 Results of quality assessment.**

| \|  \| \| --- \| |  |  |  |  |  |  |  |  |  |
| --- | --- | --- | --- | --- | --- | --- | --- | --- | --- | --- |
| Source (reference) | Selection^1^ | | | | Comparability^2^ | Outcome^3^ | | |  |
|  | Representativeness of exposed cohort | Selection of non-exposed cohort | Exposure ascertainment | No AF when investigations begin | Comparable on confounder^5^ | Outcome Assessment | Adequate follow-up(≧5y) | Loss to follow-up rate (≦10%) | Total Score^4^ |
|  | ☆ | ☆ | ☆ | ☆ | ☆☆ | ☆ | ☆ | ☆ |  |
| Hallström et al.(2019) | / | / | ☆ | ☆ | ☆☆ | ☆ | ☆ | ☆ | 7 |
| Mourtzinis et al.(2018) | / | ☆ | ☆ | ☆ | ☆☆ | ☆ | / | ☆ | 7 |
| Li et al.(2017) | ☆ | ☆ | ☆ | ☆ | ☆☆ | ☆ | ☆ | ☆ | 9 |
| Kokubo et al.(2017) | ☆ | ☆ | ☆ | ☆ | ☆☆ | ☆ | ☆ | ☆ | 9 |
| Magnussen et al.(2017) | ☆ | ☆ | ☆ | ☆ | ☆☆ | ☆ | ☆ | ☆ | 9 |
| Sciacqua et al.(2015) | / | ☆ | ☆ | ☆ | ☆☆ | ☆ | / | ☆ | 7 |
| Eryd et al.(2014) | ☆ | ☆ | ☆ | ☆ | ☆☆ | ☆ | ☆ | ☆ | 8 |
| Watanabe et al.(2011) | ☆ | ☆ | ☆ | ☆ | ☆☆ | ☆ | / | ☆ | 8 |
| Nyrnes et al.(2012) | ☆ | ☆ | ☆ | ☆ | ☆☆ | ☆ | ☆ | ☆ | 9 |
| Faye L et al.(2013) | ☆ | ☆ | ☆ | ☆ | ☆☆ | ☆ | ☆ | ☆ | 9 |
| Rosengren et al.(2009) | / | ☆ | ☆ | ☆ | ☆ | ☆ | ☆ | ☆ | 7 |
| Alonso et al.(2014) | ☆ | ☆ | ☆ | ☆ | ☆☆ | ☆ | ☆ | ☆ | 9 |
| Lee et al.(2019) | / | ☆ | ☆ | ☆ | ☆☆ | ☆ | ☆ | ☆ | 8 |
| Knuiman et al.(2014) | ☆ | ☆ | ☆ | ☆ | ☆☆ | ☆ | ☆ | ☆ | 9 |
| Psaty BM et al.(1997) | / | ☆ | ☆ | ☆ | ☆☆ | ☆ | / | ☆ | 7 |
| Alonso et al. (2013)-CHARGE-AGES | ☆ | ☆ | ☆ | ☆ | ☆ | ☆ | ☆ | ☆ | 8 |
| Alonso et al. (2013)-CHARGE-RS | ☆ | ☆ | ☆ | ☆ | ☆ | ☆ | ☆ | ☆ | 8 |
| Alonso et al. (2013)-CHARGE-CHS AA | ☆ | ☆ | ☆ | ☆ | ☆ | ☆ | ☆ | ☆ | 8 |
| Alonso et al. (2013)-CHARGE-CHS-white | ☆ | ☆ | ☆ | ☆ | ☆ | ☆ | ☆ | ☆ | 8 |
| \|  \| \| --- \| |  |  |  |  |  |  |  |  |  |
|  |  |  |  |  |  |  |  |  |  |

^1^ “Selection” part includes representativeness of cases, selection of controls, exposure ascertainment, and no death when investigation begin.

^2^ “Comparability” part includes comparable on confounders.

^3^ “Outcome” part includes outcome assessment, adequate follow-up, and loss to follow-up rate.

^4^ The total score is equal to the total number of stars.

^5^ If the studies adjusted for one individual factors among age, it is award a star. If the studies adjusting for ≥2 (sex, alcohol, BP/hypertension, heart failure, ), it is award another star.

**S**u**pplementary Table 2 Exclusion reasons**

| no | Title | Year | Reason |
| --- | --- | --- | --- |
| 1 | Lipid Profile and New-Onset Atrial Fibrillation in Patients with Acute ST-Segment Elevation Myocardial Infarction (An Observational Study in Southwest of China) | 2019 | Lack of relative risk |
| 2 | Total cholesterol variability and risk of atrial fibrillation: A nationwide population-based cohort study | 2019 | Insufficient data online |
| 3 | Predictors of 13-year risk of incident atrial fibrillation in Russian population sample of middle and elderly age | 2019 | Insufficient data online |
| 4 | High prevalence of hyperuricaemia and its impact on non-valvular atrial fibrillation: The cross-sectional Guangzhou (China) Heart Study | 2019 | cross-sectional |
| 5 | Body Mass Index Variability and Long-term Risk of New-Onset Atrial Fibrillation in the General Population: A Korean Nationwide Cohort Study | 2019 | Duplicate report on the same study population |
| 6 | Relationship between metabolic syndrome and its components and cardiovascular disease in middle-aged and elderly Chinese population: A national cross-sectional survey | 2019 | cross-sectional |
| 7 | Effect of the variability of blood pressure, glucose, total cholesterol, and body mass index on risk of atrial fibrillation in a healthy population | 2019 | Insufficient data online |
| 8 | The impact of metabolic syndrome on the incidence of atrial fibrillation: A nationwide longitudinal cohort study in South Korea | 2019 | Duplicate report on the same study population |
| 9 | Clinical study for oxidative stress and lipid profile levels in patients with cardiac arrhythmias | 2019 | Insufficient data online |
| 10 | Metabolically Healthy Obesity and Risk for Atrial Fibrillation: The HUNT Study | 2019 | Insufficient data online |
| 11 | Atrial Fibrillation Manifestations Risk Factors and Sex Differences in a Population-Based Cohort (From the Gutenberg Health Study) | 2018 | cross-sectional study |
| 12 | Change in lipid profile and risk of new-onset atrial fibrillation in patients with chronic heart failure A 3-year follow-up observational study in a large Chinese hospital | 2018 | Insufficient data online |
| 13 | Association between blood lipid profiles and atrial fibrillation: A case-control study | 2018 | case-control study |
| 14 | Epidemiological Characteristics of Atrial Fibrillation in Southern China: Results from the Guangzhou Heart Study | 2018 | cross-sectional study |
| 15 | High-Density Lipoprotein Cholesterol (HDL-C) Levels Independently Correlates with Cardiac Arrhythmias and Atrial Fibrillation | 2018 | Lack of relative risk |
| 16 | Atrial fibrillation: Prevalence and cross-sectional determinants in a population sample of 9255 participants | 2017 | cross-sectional |
| 17 | Association of pre-ablation level of potential blood markers with atrial fibrillation recurrence after catheter ablation: A meta-analysis | 2017 | meta-analysis |
| 18 | The association between insulin resistance and atrial fibrillation: A cross-sectional analysis from SPRINT (Systolic Blood Pressure Intervention Trial) | 2017 | cross-sectional study |
| 19 | The associations between atrial fibrillation and parameters of nutritional status assessment in the general hospital population -A cross-sectional analysis of medical documentation | 2017 | cross-sectional study |
| 20 | The CHADS2 and CHA2DS2-VASc scores predict atrial fibrillation in dyslipidemic individuals: Role of incorporating low high-density lipoprotein cholesterol levels | 2017 | Lack of relative risk |
| 21 | Are cardiovascular risk factors also associated with the incidence of atrial fibrillation ?: A systematic review and field synopsis of 23 factors in 32 population-based cohorts of 20 million participants | 2017 | systematic review |
| 22 | Ideal cardiovascular health metrics on the prevalence of atrial fibrillation in Chinese population: A cross-sectional study | 2016 | cross-sectional study |
| 23 | Usefulness of the Electrocardiographic P-Wave Axis as a Predictor of Atrial Fibrillation | 2016 | Insufficient data online |
| 24 | Association of lipid-related genetic variants with the incidence of atrial fibrillation: The AFGen consortium | 2016 | Duplicate report on the same study population |
| 25 | Prevalence and associating factors of atrial fibrillation in patients with hypertension: A nation-wide study | 2016 | cross-sectional |
| 26 | Prevalence of atrial fibrillation and its risk factors in rural China: A cross-sectional study | 2015 | cross-sectional study |
| 27 | Racial Differences in Incidence and Clinical Course of Atrial Fibrillation and What Remains to be Investigated | 2015 | Review |
| 28 | Prevalence of Atrial Fibrillation and Relation to Echocardiographic Parameters in a Healthy Asymptomatic Rural Korean Population | 2015 | cross-sectional study |
| 29 | HDL cholesterol and high-sensitive troponin T as predictives biomarkers of atrial fibrillation after heart surgery | 2015 | Lack of relative risk |
| 30 | Atrial fibrillation in patients admitted to coronary care units in western Sweden - Focus on obesity and lipotoxicity | 2015 | Insufficient data online |
| 31 | Incident Atrial Fibrillation Hazard in Hypertensive Population: A Risk Function from and for Clinical Practice | 2015 | Insufficient data online |
| 32 | Racial differences in incident atrial fibrillation among hypertensive patients during antihypertensive therapy | 2014 | Insufficient data online |
| 33 | Uric acid is associated with future atrial fibrillation: An 11-year follow-up of 6308 men and women - the Tromsø study | 2014 | Insufficient data online |
| 34 | Paradoxical association of lipoprotein measures with incident atrial fibrillation | 2014 | Insufficient data online |
| 35 | Serum cholesterol levels and postoperative atrial fibrillation | 2014 | Insufficient data online |
| 36 | Metabolic syndrome and atrial fibrillation in patients with essential hypertension | 2013 | Insufficient data online |
| 37 | Inflammatory biomarkers as risk factors for future atrial fibrillation. An eleven-year follow-up of 6315 men and women: the Tromso study | 2012 | Insufficient data online |
| 38 | Impact of alcohol habits and smoking on the risk of new-onset atrial fibrillation in hypertensive patients with ECG left ventricular hypertrophy: The LIFE Study | 2012 | Insufficient data online |
| 39 | Association between lipid profile and risk of atrial fibrillation | 2011 | Duplicate report on the same study population |
| 30 | Association of serum uric acid with incident atrial fibrillation (from the Atherosclerosis Risk in Communities [ARIC] study) | 2011 | Insufficient data online |
| 31 | Chronic kidney disease and prevalent atrial fibrillation: The Chronic Renal Insufficiency Cohort (CRIC) | 2010 | No RR |
| 42 | Atrial fibrillation is a possible marker of frailty in hospitalized patients: Results of the GIFA Study | 2010 | Insufficient data online |
| 43 | Metabolic syndrome and incidence of atrial fibrillation among blacks and whites in the Atherosclerosis Risk in Communities (ARIC) Study | 2010 | Insufficient data online |
| 44 | Metabolic syndrome and risk of development of atrial fibrillation: The Niigata preventive medicine study | 2008 | Insufficient data online |
| 45 | Lipoprotein(a) and lipid profile in patients with atrial fibrillation | 2006 | Insufficient data online |
| 46 | Prevalence of and risk factors for atrial fibrillation in Korean adults older than 40 years | 2005 | Insufficient data online |
| 47 | Hospitalizations for atrial fibrillation in the general male population: Morbidity and risk factors | 2001 | No RR |
| 48 | Cholesterol paradox in patients with paroxysmal atrial fibrillation | 1999 | case-control study |
